# Point-of-care umbilical arterial lactate and newborn outcomes in a low resource setting: cohort study

**DOI:** 10.1186/s13104-018-3598-9

**Published:** 2018-07-16

**Authors:** George Kassim Chilinda, Luis Aaron Gadama, William Stones

**Affiliations:** 1 Department of Obstetrics and Gynaecology (Ethel Mutharika Maternity Wing), Kamuzu Central Hospital, Lilongwe, Malawi; 2 Department of Obstetrics and Gynaecology, College of Medicine, Blantyre, Malawi; 3 Departments of Public Health and Obstetrics & Gynaecology, College of Medicine, Blantyre, Malawi

**Keywords:** Birth asphyxia, Fetal distress, Hyperlactatemia, Newborn care, Neonatal death, Malaria

## Abstract

**Objective:**

Birth asphyxia contributes substantially to the burden of intrapartum stillbirth and neonatal mortality in resource limited countries. We investigated clinical correlates and neonatal outcomes of lactate analysis of umbilical arterial cord blood in a large referral maternity unit in Malawi using a point-of-care test (Lactate Xpress, Nova Biomedical, Runcorn, UK) and examined maternal and neonatal characteristics and outcomes.

**Results:**

There were 389 live births and 12 intrapartum stillbirths during the study. The median umbilical arterial lactate concentration was 3.4 mmol/L (interquartile range 2.6–4.9). Umbilical arterial lactate concentrations among the 45 babies admitted for special neonatal care were above 5 mmol/L in 16/45 (36%) of cases, with no fatality below 13 mmol/L. A positive malaria rapid diagnostic test was associated with hyperlactatemia (p < 0.05). In receiver-operator characteristic (ROC) analysis using a lactate cutoff of 5 mmol/L, areas under the curve were 0.72 (95% CI 0.66–0.79) and 0.64 (95% CI 0.58–0.69) for the Apgar score at 1 and 5 min respectively. This approach can identify safely those newborns that are unlikely to require additional monitoring. Scale-up implementation research in low resource country referral units is needed. The influence of malaria on neonatal hyperlactatemia requires further exploration.

## Introduction

Alongside efforts to strengthen maternal and fetal safety through detection of problems in the antenatal period, monitoring during labour and delivery [1], and ensuring access to immediate newborn resuscitation where needed, a need has been recognised for tools to support clinical decision making and care. While Apgar scoring remains the mainstay of clinical assessment and has been used extensively in outcome studies, its precision may be limited by the skill and experience of staff. Furthermore, some newborns with significant hypoxia and acidaemia may pass unrecognised owing to a normal Apgar score but suffer adverse consequences later such as poor feeding, hypoglycaemia, and seizures; by contrast, scores may be low from causes other than birth asphyxia. To improve the diagnosis of birth asphyxia, acid–base analysis of umbilical cord blood has been deployed in maternity units in high resource countries either for all births or in ‘high risk’ cases according to national guidelines [2]. Umbilical cord lactate estimation is attractive as a simpler alternative to blood gas analysis, using either a hospital chemistry analyser or a point-of-care device. Its validity has been confirmed in a systematic review [3] that also emphasised the need for studies in low- and middle-income countries with a higher burden of newborn complications. A point-of-care approach has recently been reported from a middle-income country setting, using lactate as a guide to clinical care and for feedback to clinicians about the accuracy of their intrapartum monitoring and its interpretation [4].

In our extremely resource-limited maternity service context, we aimed to assess the potential of umbilical arterial lactate analysis using a point-of-care test alongside clinical assessment with Apgar scoring, taking maternal risk factors into account, to identify newborns at risk of complications, so as to focus the available neonatal care resources on those most in need.

## Main text

This was a cohort study of all live births that occurred at the Queen Elizabeth Central Hospital, Blantyre, Malawi in a 2-week period during April 2017. This is the largest referral maternity unit in southern Malawi and provides both secondary and tertiary referral services residents of Blantyre residents and the seven surrounding districts. It has a labour ward with 24 delivery beds and has an average of 720 deliveries per month. For the present study all women admitted in labour at an estimated gestational age of 28 weeks or more were included, whether they went on to deliver in the labour ward or by Caesarean section in the operating theatre. Gestational age in this setting is often uncertain was but taken as the best available estimate from the date of the last menstrual period where this was recalled, fundal height documented at the first antenatal attendance, or an ultrasound scan in the small number of cases where this had been performed up to 24 weeks. Deliveries before arrival at the maternity unit were excluded as were those of mothers who arrived already actively pushing in the second stage of labour.

For live births, routine procedures for newborn care were followed including recording of 1- and 5-min Apgar scores with clinical resuscitative measures taken where required. Babies born in the hospital with a birth weight below 2.5 kg are routinely assessed for either special care nursery admission, ‘kangaroo mother care’ or tracking of breastfeeding and weight gain in the routine postnatal ward or at home. We sampled umbilical cord arterial blood from a segment of cord isolated between clamps analysed lactate concentration within 15 min of each birth using a point-of-care test (Lactate Xpress, Nova Biomedical, Runcorn, UK). The test strips have been reported to show excellent correlation of lactate results with those from a conventional laboratory analyser when tested using umbilical cord blood and also showed low analytical imprecision with a coefficient of variation of 5.5% [5]. Postnatal blood samples from mothers were analysed at the bedside for syphilis, malaria and haemoglobin. Where the maternal HIV status was unknown or documented as negative during the antenatal period, a repeat test was offered. Mothers’ health records were scrutinised to identify antenatal health issues, labour, delivery and postnatal details, while neonatal special care nursery records were used to confirm neonatal outcomes known up to the point of discharge. Autopsy was not available following neonatal deaths, so all diagnoses were based on clinical findings and available laboratory results. The sample size was based on scrutiny of delivery unit registers that suggested a possible birth asphyxia rate between 10 and 27%. Samples of 162 and 348 respectively would have 80% power to identify these rates with an error margin of 3%.

Data were entered in Microsoft Excel and analysed using Stata version 14 using Chi square and ‘t’ tests as appropriate taking 5% probability as statistically significant. Maternal and neonatal variables were tabulated against normal or elevated umbilical cord arterial lactate findings. For multiple births, only the firstborn lactate results were included in analyses of maternal age, parity and educational status. Receiver-operator characteristic (ROC) curves were constructed to examine the relationship between umbilical arterial lactate and the 1- and 5-min Apgar scores.

Written informed consent was obtained on admission to the maternity unit. The study was approved by the Hospital Director and by the College of Medicine Research Ethics Committee, reference P.06/16/1963.

### Results

During the study period 380 eligible participants were enrolled leading to 389 live births and 12 intrapartum stillbirths. 87 (21.7%) of newborns had birth weights below 2500 g. Neonatal special care admission was arranged for 45 (11.5%) newborns based on low Apgar score or clinical suspicion. There were six early neonatal deaths, representing an in-hospital perinatal mortality rate of 44.9/1000 live births (95% CI 27–70). There were no maternal deaths. Almost all mothers had attended for at least one antenatal visit and 43% had attended on four or more occasions. 10% of mothers had no education while 8% had received tertiary education. All mothers had been tested for HIV: 12.1% were positive, all of whom were on antiretroviral therapy by the time of delivery according to national guidelines.

**Fig. 1:**
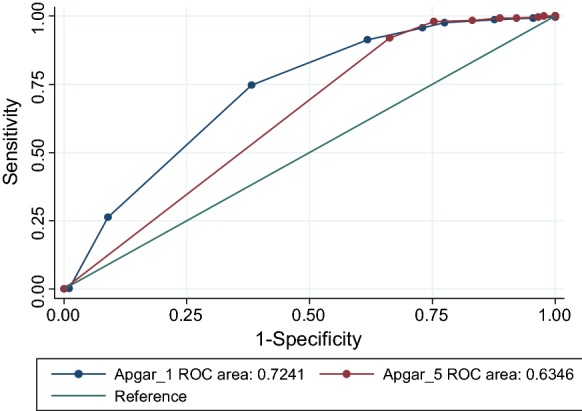
Receiver-operator characteristics (ROC) plot of 1- and 5-min Apgar scores using an umbilical arterial lactate concentration cut-off of 5 mmol/L

The median umbilical cord arterial lactate concentration was 3.4 mmol/L (interquartile range 2.6–4.8). Clinical correlates, laboratory findings and their associations with hyperlactatemia (above 5 mmol/L) are shown in Table 1. Lactate concentration did not differ by birth order in twin and triplet deliveries. Malaria rapid diagnostic tests were positive in seven cases. Of these, umbilical cord arterial lactate results were below 5 mmol/L in three and above this threshold in four cases, with hyperlactatemia of 5.9, 6.7, 7.5 and 12.2 mmol/L respectively. All seven mothers were recorded as having received at least one dose of intermittent presumptive antimalarial therapy in the antenatal period. In receiver-operator characteristic (ROC) analysis using a lactate cutoff of 5 mmol/L, areas under the curve were 0.72 (95% CI 0.66–0.79) and 0.64 (95% CI 0.58–0.69) for the Apgar score at 1 and 5 min respectively (Fig. 1). Using a lower lactate cutoff of 3.2 mmol/L the areas under the curve were lower, at 0.62 and 0.56 respectively.

**Table 1:** Clinical and laboratory findings by umbilical cord arterial hyperlactatemia (5 mmol/L or more)

Participant status	Cord lactate (5 mmol/L or more)		N	p-value
	< 5 mmol/L	5 mmol/L +		
Age (mean, std err.)	26.1 (0.40)	25.1 (0.72)	368	0.223
Parity (no, %)				
0	88 (31.2)	30 (34.9)	368	0.126
1–2	114 (40.4)	41 (47.7)		
3+	80 (29.1)	15 (17.4)		
Maternal education (no, %)				
None	30 (10.6)	8 (9.3)	368	0.086
Primary	107 (37.9)	43 (50.0)		
Secondary	125 (44.3)	26 (30.2)		
Tertiary	20 (7.1)	9 (10.5)		
Booking weight, kg (mean, std err.)	63.5 (0.72)	62.9 (1.27)	334	0.688
Hypertension (no, %)	27 (9.0)	9 (10.0)	389	0.781
Antepartum urinary tract infection (no, %)	21 (7.0)	7 (7.8)	389	0.808
Birth from multiple pregnancy (no, %)	32 (10.7)	8 (8.9)	389	0.619
Birth after antepartum haemorrhage (no, %)	6 (2.0)	4 (4.4)	389	0.200
Birth after pre-labour rupture of membranes	19 (6.4)	4 (4.4)	389	0.501
Meconium stained liquor during labour (no, %)	26 (8.7)	14 (15.6)	389	0.060
Vaginal delivery (no, %)	185 (61.9)	48 (53.3)	389	0.147
Birthweight, g (mean, std err)	2847 (31.6)	2900 (57.6)	389	0.422
Maternal investigations				
Haemoglobin, g/dL (mean, std err)	11.2 (0.09)	11.1 (0.17)	389	0.462
HIV positive (no, %)	40 (13.7)	6 (6.7)	381	0.078
Syphilis rapid test positive (no, %)	4 (1.3)	1 (1.1)	388	0.865
Malaria rapid test positive (no, %)	3 (1.0)	4 (4.4)	389	0.031

Among the six early neonatal deaths, umbilical arterial lactate concentrations were above 13 mmol/L in five cases of which four succumbed soon after birth and before admission to the special care neonatal unit. In one further case, early neonatal death occurred before neonatal unit admission and the lactate concentration was 4.0 mmol/L, suggesting that the fatality may have been due to a cause other than birth asphyxia.

Umbilical arterial lactate concentrations among the 45 babies admitted for special neonatal care were above 5 mmol/L in 16/45 (36%) of cases and above 11 mmol/L in 6/45 (13%). No neonatal unit fatality occurred where the lactate concentration was below 13 mmol/L and there were no early neonatal deaths where the 5 min Apgar score had been 6 or more.

### Discussion

We have identified the range of umbilical cord arterial lactate concentrations in our low-resource referral hospital setting and found the point-of-care test to be feasible. We have identified the lactate levels at which good newborn outcomes can be anticipated despite an initial low Apgar score. We show that Apgar scoring has performed well in this setting at least in the context of this study, as no newborns with normal 5 min Agpar suffered complications and especially early neonatal death. We consider that the main value of umbilical cord arterial lactate estimation in settings such as ours will be to identify those newborns with a low Apgar score but not at big risk of complications. For service organisation, this will allow them to be monitored in a lower-intensity manner, enabling staff and resources to be concentrated on those in greater need of neonatal intensive care or supportive interventions. To date, serial lactate estimation has not been widely used in neonatal high or intensive care as a prognostic indicator, unlike its well established role in adult critical care. Our study provides a basis from which to extend research from the immediate peripartum period using cord samples, to examine the potential for its use in early neonatal care in low resource settings.

Our finding of a significantly higher proportion of cord hyperlactatemia associated with a positive malaria rapid test is a novel finding although we note the number of malaria-positive cases in our cohort was small. The work was undertaken towards the end of the main malaria transmission season in the country. Mothers who were positive on the rapid test were not clinically unwell and the clinical and pathophysiological implications of cord hyperlactatemia are therefore uncertain. The use of point-of-care lactate estimation has been studied in the context of childhood malaria [6] and there is clearly a need to explore both the mechanistic implications and potential clinical applications of umbilical cord blood chemistry in malaria-prevalent settings.

While under-five mortality has declined substantially in many low resource countries over the past 15 years, considerable challenges remain in proving safe maternity care, as evidenced by high rates of maternal and neonatal death even in countries where facility-based delivery has become the norm such as Malawi [7]. In particular, intrapartum fetal death and birth asphyxia remain a major problem owing to lack of monitoring during labour and the immediate newborn period [1]. While much can be achieved by the consistent application of clinical skills and triage protocols and indeed this is supported by national-level standards and training [8], there is in important gap in relation to rapid and precise identification of neonates who are at risk of complications, or those who require a lower intensity of postnatal observation. Economic analysis will be needed to examine the trade-off between cost of point-of-care lactate testing including the cost of consumables, versus the resources potentially saved in additional days in special care nursery. Implementation research is needed to take this approach to scale, for example a selective strategy of clamping a segment of cord, sampling the blood and testing only if the 5 min Apgar score is below 7 or if there is other suspicion regarding the neonatal condition. Potential related benefits of maternity units having ready access to point-of-care lactate analysis include its use for assessing the maternal condition [9, 10]. The option of laboratory-based lactate analysis is unlikely to be a viable alternative in settings such as ours owing to slow turnaround time and challenges with consistent availability of reagents and quality control.

## Limitations

The range of lactate results seen here is consistent with that reported in the large population study that defined percentiles associated with normal newborn outcomes [11]. That study identified modest but measurable effects of gestational age on cord lactate levels and a further study by the same group [12] noted similar influences of the time taken for cord clamping. Gestational age is difficult to assess reliably in our setting and it is impractical to specify precise timing for sampling in relation to cord clamping in busy units with staffing challenges such as ours. This could potentially affect lactate results but the effects reported are modest in relation to the normal range and are unlikely to result in serious mis-classification of hyperlactatemia using a 5 mmol/L cutoff.

## Abbreviation

ROC: receiver-operator characteristics
